# Signal Exchange through Extracellular Vesicles in Neuromuscular Junction Establishment and Maintenance: From Physiology to Pathology

**DOI:** 10.3390/ijms20112804

**Published:** 2019-06-08

**Authors:** Serena Maggio, Paola Ceccaroli, Emanuela Polidori, Andrea Cioccoloni, Vilberto Stocchi, Michele Guescini

**Affiliations:** Department of Biomolecular Sciences, University of Urbino Carlo Bo, Via I Maggetti, 26, 61029 Urbino, Italy; serena.maggio@uniurb.it (S.M.); paola.ceccaroli@uniurb.it (P.C.); emanuela.polidori@uniurb.it (E.P.); a.cioccoloni@campus.uniurb.it (A.C.); vilberto.stocchi@uniurb.it (V.S.)

**Keywords:** neuromuscular junction, extracellular vesicles, exosomes, miRNA, morphogens, Wnts, motor neuron disorders

## Abstract

Neuromuscular junction (NMJ) formation involves morphological changes both in motor terminals and muscle membrane. The molecular mechanisms leading to NMJ formation and maintenance have not yet been fully elucidated. During the last decade, it has become clear that virtually all cells release different types of extracellular vesicles (EVs), which can be taken up by nearby or distant cells modulating their activity. Initially, EVs were associated to a mechanism involved in the elimination of unwanted material; subsequent evidence demonstrated that exosomes, and more in general EVs, play a key role in intercellular communication by transferring proteins, lipids, DNA and RNA to target cells. Recently, EVs have emerged as potent carriers for Wnt, bone morphogenetic protein, miRNA secretion and extracellular traveling. Convincing evidence demonstrates that presynaptic terminals release exosomes that are taken up by muscle cells, and these exosomes can modulate synaptic plasticity in the recipient muscle cell in vivo. Furthermore, recent data highlighted that EVs could also be a potential cause of neurodegenerative disorders. Indeed, mutant SOD1, TDP-43 and FUS/TLS can be secreted by neural cells packaged into EVs and enter in neighboring neural cells, contributing to the onset and severity of the disease.

## 1. Introduction

The neuromuscular junction (NMJ) is a chemical synapse formed between motoneurons and skeletal muscles and is covered by Schwann cells (SCs). NMJ is essential for our physical mobility and daily life.

NMJ translates the electrical impulses delivered by the motoneuron into action potentials in the juxtaposed muscle fiber. The conversion of electrical into chemical signals relays on acetylcholine (ACh) release from presynaptic terminals, as well as high-density clustering of nicotinic acetylcholine receptors (nAChRs) on the postsynaptic muscle membrane [1,2].

Vertebrate NMJs are the best-studied peripheral synaptic units. During vertebrate embryonic development, motor nerves penetrate peripheral regions to reach myotubes. Subsequently, motoneuron axons innervate skeletal muscle at the end-plate band, a discrete central region where axon terminals branch into synaptic varicosities apposed to foldings of the muscle membrane, the junctional folds [3,4,5,6].

NMJ formation is a multi-step process that requires a coordinated integration of both anterograde and retrograde signals and involves morphological changes both in presynaptic motor terminals and postsynaptic muscle fiber membrane. Presynaptic active zone differentiation is accompanied by morphological changes in motor terminals that begin to accumulate synaptic vesicles containing acetylcholine, neurotransmitter receptors and other presynaptic components.

In turn, postsynaptic differentiation is characterized by an enrichment in AChRs, and acetylcholinesterase (AChE), which rapidly degrades acetylcholine and small invaginated fractions of the muscle membrane to shape the mature NMJ [7]. Before the neuromuscular junction takes place, aneural AChRs are first clustered in a central and prospective synaptic region of the muscle, a process called muscle pre-patterning [8]. After the interaction between motor axon and muscle, AChR clusters form at postsynaptic regions exactly opposed to the sites of nerve contact. These mature clusters (also called neural AChR clusters) form by recruiting aneural AChR clusters as well as accumulating newly synthesized AChRs [7].

Motoneurons and muscle cells develop independently, but their mutual interactions contribute to NMJ formation. Indeed, a growing body of evidence suggests that NMJ development requires extensive communication among the three components of the synapse: presynaptic motoneurons, postsynaptic muscle fibers and SCs. For example, the current working model indicates that the apposition of pre- and postsynaptic terminals could be driven by the signal exchange between motoneurons and target muscle fibers. Indeed, it has been demonstrated that both nerves and muscles secrete signaling proteins acting at both sides of the NMJ to exert positive and negative effects on the differentiation of pre- and postsynaptic terminals [7,9,10,11].

For example, motoneuron terminals secrete factors such as agrin that act to concentrate AChRs at the NMJ by promoting the activation of signaling pathways involved in NMJ structure and function. Motoneurons also release ACh, a negative signal that suppresses this machinery, to eliminate supernumerary AChR clusters from extrasynaptic regions. On the other side, the innervated muscle releases retrograde factors involved in AChR transport to the post-junctional membrane as well as AChR clustering and stability [11].

Despite intense studies, the molecular mechanisms that regulate and stabilize AChR clustering remain unclear. Cumulative evidence obtained from different model organisms has demonstrated that signaling molecules that act during early development, such as Wnts, bone morphogenetic proteins (BMPs) and miRNAs, regulate crucial events that lead to the formation and maintenance of proper neuronal connections. This review is focused on the involvement of extracellular vesicles (EVs) in carrying these signals in physiological and pathological conditions. Before dealing with the recent evidence on the role of EVs in mediating signal exchange at the NMJ, the signal pathways mainly involved in the establishment and function of the NMJ are introduced.

## 2. Signaling Pathways in the Establishment and Function of the Neuromuscular Junction

Muscle-specific kinase (MuSK) is a member of the receptor tyrosine kinase superfamily; it is required for both aneural and neural AChR cluster formation [12]. The activation mechanism of MuSK is complex, involving at least three other key proteins: agrin, derived from motoneurons, Lrp4 (low-density lipoprotein receptor-related protein 4) and Dok7, which are expressed in the plasma membrane and cytoplasm of muscle myotubes, respectively [13].

In greater detail, MuSK can form a receptor complex with Lrp4 acting as a central scaffold that orchestrates all steps of NMJ formation and maintenance from the postsynaptic side [14,15]. Mutant mice lacking agrin, Lrp4 or MuSK do not form NMJs, indicating their critical role in NMJ formation. In addition to Lrp4, MuSK requires Dok7 for activation. In response to stimulation signals, Dok7 becomes a substrate of MuSK and is phosphorylated on Tyr396 and Tyr406. In turn, phosphorylated Dok7 recruits the adaptor proteins Crk and Crk-L, which play a role in NMJ formation [16]. Dok7-deficient mice show neither muscle pre-patterning of AChRs nor NMJ formation. Given that mutations in the human *Dok7* gene result in congenital myasthenia (*Dok7* myasthenia) [17], Dok7 would be required not only for the formation but also for the maintenance of NMJs [16].

The glycoprotein agrin plays an essential role in NMJ formation; it is synthesized in motoneuron’s cell body, transported along the axon and secreted into synaptic clefts. Once released, agrin binds to Lrp4, activating MuSK [18], which leads to AChR clustering in the postsynaptic membrane. Agrin is sufficient to induce ectopic AChR clusters in adult muscles [19] and to elicit the formation of a postsynaptic apparatus in denervated muscles [20].

Moreover, experimental data show that Lrp4 interacts with MuSK even in the absence of agrin. This basal interaction, along with the action of Dok7, is sufficient for the partial activation of MuSK, which seems to be important for the pre-patterning of AChRs in myotubes before innervation [10].

The extracellular region (ectodomain) of Musk is characterized by sequence and structural similarities to the cysteine-rich domain (CRD) of Frizzled (Fz) proteins, the receptors for Wnts. In agreement with this finding, it has been suggested that Wnts may regulate NMJ formation through MuSK/Lrp4 signaling [21,22].

Wnts act as morphogens to regulate crucial events of cell fate and development; in the formation of neuronal circuits, Wnts regulate axon guidance, dendritic development, synaptogenesis and synaptic function [23,24,25]. There are multiple Wnt isoforms (19 in mice and humans) transduced by Fz receptors and co-receptors such as Lrp5 and Lrp6 [26]. Three main Wnt pathways can be activated by Wnt ligands: (i) the Wnt canonical pathway; (ii) the Wnt planar cell polarity (PCP) pathway; and (iii) the Wnt calcium-dependent pathway.

In the canonical pathway, the binding of Wnts to Fz receptors and Lrp5/6 activates dishevelled (Dvl), which inhibits glycogen synthase kinase-3 β (Gsk-3β), leading to the disassembly of axin destruction complex (formed by Gsk-3β, axin, adenomatous polyposis coli protein (APC) and β-catenin). This results in the inhibition of β-catenin phosphorylation and its accumulation in the cytoplasm [27,28,29,30], followed by β-catenin translocation to the nucleus to regulate gene expression.

In PCP signaling, Fz receptors activate a cascade of the small GTPases RAC1 and RHOA, which in turn activate c-Jun N-terminal kinase (JNK) and Rho kinase (ROCK) as downstream effectors that control cytoskeleton rearrangements and gene expression [31].

In the Wnt–Ca^2+^ signaling pathway, Fz activation leads to increased intracellular Ca^2+^, which can activate protein kinase C (PKC) and calcium/calmodulin-dependent protein kinase II (CaMKII) as well as the nuclear import of the transcription factor nuclear factor of activated T cells (NF-AT).

Among the Wnt isoforms, it has been reported that Wnt4, 9a and 11 can bind the MuSK complex through CRD and/or Lrp4 to promote AChR clustering [22,32,33]. In contrast, Wnt3a disperses AChR clusters by repressing the expression of Rapsyn, a scaffold protein essential for the anchoring of AChRs [34]. In addition, several other Wnts negatively (Wnt7a, 8a and 10b) or positively (Wnt3 and 10b) modulate aneural or agrin-induced AChR clusters in a MuSK-independent manner [4,16].

Wnt canonical β-catenin dependent and non-canonical PCP and calcium signaling branches are intersecting pathways for successful NMJ formation. Dvl1 acts at the crossroad of the Wnt/ β-catenin and Wnt/PCP pathways, and it interacts with MuSK and regulates AChR clustering in muscle cells [35]. The Wnt canonical pathway in muscle translocates β-catenin to the nucleus and it drives the expression of a retrograde signaling that controls motor axon outgrowth within the target field and presynaptic differentiation. For example, Slit2, a well-known axon guidance cue, has been recently identified as one of the β-catenin-dependent retrograde factors [36]. In the PCP pathway, Vangl2 accumulates in the synapse-rich region and leads to AChR clustering and/or stabilization. Alterations of the Wnt canonical pathway or the Vangl2-dependent PCP pathway induce similar NMJ defects; both pathways would then initiate muscle pre-patterning and contribute to nerve/muscle target recognition and NMJ differentiation/maturation [25].

Recent studies, mainly in *Drosophila*, have also revealed crucial functions for the bone morphogenetic protein (BMP) signaling pathway, which has been described as the major retrograde pathway promoting NMJ growth [37]. BMP signaling involves the formation of heteromeric complexes of two types of transmembrane receptors, BMPRI and BMPRII. For example, upon BMP-2 binding, the activated BMPRI phosphorylates the proteins Smad-1, -5 or -8, which in turn form heteromeric complexes with the common mediator Smad-4. Once activated, Smad complexes translocate into the nucleus to regulate the transcription of specific target genes.

It has been reported that agrin can bind BMP-2 and -4, thus increasing the mRNA expression levels and the immunoreactivity of BMP-4 in differentiated C2C12 muscle cells [38]. In line with these data, BMPs expressed by muscle cells exert autocrine effects, but could also be involved in the modulation of motor neuron behavior. This hypothesis is consistent with the crucial role that the retrograde secretion of the BMP ligand glass bottom boat (Gbb) plays on the nerve terminal development and NMJ establishment in *Drosophila* [39]. In the NMJ development and growth, in vivo evidence points out an anterograde action of Wnt signals on postsynaptic differentiation, whereas BMP ligands likely act through retrograde mechanisms to induce presynaptic effects.

## 3. Secretion and Transport of Morphogens via Extracellular Vesicles

Due to post-translational lipid modifications, Wnts appear paradoxically ill-suited to free diffusion in the hydrophilic extracellular environment. Indeed, one of the mechanisms for transporting lipophilic ligands is to load them onto membranous vesicles. Recently, exosomes have emerged as potent carriers for Wnt secretion and extracellular traveling [40,41,42,43].

The first evidence of Wnt loading onto lipid-based vehicles comes from the expression studies of the GPI-anchored GFP in the *Drosophila*; when GPI–GFP expression was driven in Wingless-expressing cells, Wg (Wingless, the fly orthologue of mouse Wnt1) was found to co-localize within these migrating GFP-positive particles, called argosomes [44].

Virtually all cells release different types of extracellular vesicles (EVs), which can be taken up by nearby or distant cells, modulating their activity. Therefore, EVs can act as intercellular mediators in many physiological and pathological situations, including development, physiological communication, immune response, cancer progression and metastasis, as well as cardiovascular and neurodegenerative diseases [45].

These vesicles have received different names over the years (i.e., nanoparticles, microparticles, argosomes, etc.), but today are often collectively referred to as EVs. According to the International Society for Extracellular Vesicles, three main types of EVs have been described based on their mechanism of release and size: exosomes are less than 150 nm in diameter, while microvesicles/shedding particles and apoptotic bodies are both considered to be larger than 100 nm [46].

Exosomes are small-membrane vesicles of endocytic origin that are secreted by many cells. These vesicles are formed by the inward budding of the limiting membrane of late endosomes and accumulate as intraluminal vesicles (ILVs) inside multivesicular bodies (MVBs). Stimulation of cells induces the fusion of the limiting membrane of the MVBs with the plasma membrane and the secretion of the ILVs, which then are termed as exosomes [47].

The last two types of vesicles are released directly from the plasma membrane in living and dying cells, respectively. Shedding microvesicles originate by outward budding from the surface of the plasma membrane, followed by fission [48].

Initially, exosomes were associated with a mechanism involved in the elimination of unwanted material [49]. However, subsequent experimental evidence demonstrated that exosomes, and more generally, extracellular vesicles, play a key role in intercellular communication [50]., Recent data also suggest that exosomes represent an alternative way of releasing waste products to maintain cellular homeostasis [51].

Today, EVs have gained particular attention owing to their fundamental role in cell-to-cell communication by transferring genetic information between cells. Indeed, EVs can carry proteins, lipids, DNA, mRNA and microRNA to target cells, and each of them may be involved in changes in target-cell phenotype [52].

Recent evidence from *D. melanogaster* showed the relevance of EVs in neuron–muscle communication, demonstrating the release and transport of Wg via exosomes in vivo in the larval NMJ. Furthermore, several studies have now extended this role of exosomes in Wnt signaling to human and mouse models; in detail, a portion of Wnt3a and Wnt5a was found to co-fractionate with exosomal markers in murine fibroblasts and human Caco-2 cells, respectively. Of note, these studies demonstrated that Wnts carried by exosomes could activate Wnt-responsive reporters in cell culture [42,43].

Regarding the *Drosophila* larval NMJ, one of the best-characterized models, the release of Wg also occurs in association with exosomes through its binding to the transmembrane protein Evi (also known as Wntless), which is present in MVBs. Evidence for Evi-dependent loading of Wg in exosomes comes from expressing Evi-GFP in motoneurons, which led to the appearance of Evi in the subsynaptic reticulum in muscle tissue; on the other hand, Evi downregulation in motoneurons resulted in lower levels of endogenous Evi in muscles and stunted NMJs. The secretion of Evi-containing exosomes is inhibited by lowering extracellular [Ca^2+^]. Furthermore, co-localizations of Cd63 with Wg were observed both within the MVB and outside the source cell. In line with the above-reported data, it was recently demonstrated that exosomes released by C2C12 muscle cells can induce motoneuron development and branching in NSC-34 cells [53].

**Figure 1:**
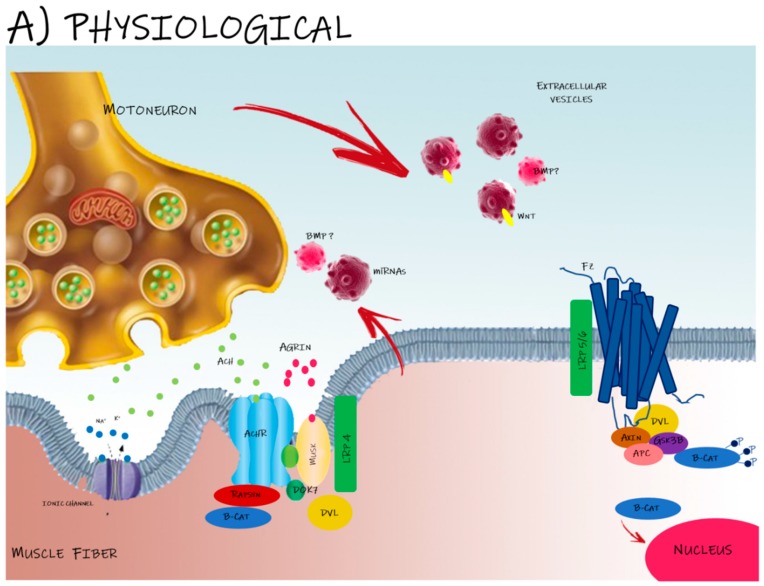

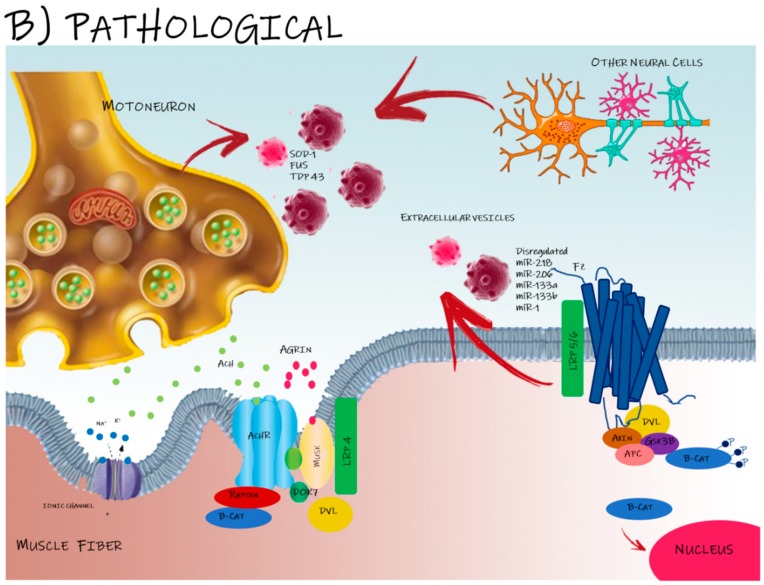
The role of extracellular vesicles (EVs) in the motoneuron–muscle communication. (**A**) Neuromuscular junction (NMJ) formation involves morphological changes both in presynaptic motor terminals and postsynaptic muscle membrane. Mounting evidence shows that EVs carry morphogens, such as Wnts and BMPs, and several miRNAs, suggesting their involvement in the instauration and maintenance of NMJ. In the extracellular milieu, soluble and EV-carried signals can activate agrin–Lrp4–MuSK signaling required for both aneural and neural AChR clustering. MuSK forms a receptor complex with Lrp4 acting as a central scaffold that orchestrates all steps of NMJ formation and maintenance in the postsynaptic side. Once released by the motoneuron, agrin together with Wnt bind their target receptors, activating MuSK tyrosine kinase which leads to AChR clustering in the postsynaptic membrane. Lrp4–MuSK interaction, along with the action of Dok7, is sufficient for the partial activation of MuSK, which is important for pre-patterning of AChRs in myotubes before innervation. Wnts can also bind to Fz receptors and Lrp5/6, activating Dvl, which inhibits Gsk-3β and leads to the disassembly of axin destruction complex (formed by Gsk-3β, axin, APC and β-catenin). Wnt pathway activation results in the inhibition of β-catenin phosphorylation and its translocation into the nucleus to regulate target genes. (**B**) Dysregulation of signal exchange mediated by EVs at the NMJ in motoneuron disorders. Familiar amyotrophic lateral sclerosis (FALS) disease is caused by mutations in the prion-like domains of SOD1, TDP43 and FUS/TLS proteins. These mutations induce protein aggregation and the formation of insoluble granules which accumulate into the neuronal cells, leading to cell death and subsequently to denervation and NMJ degradation. The transmission of these misfolded proteins is mediated by EVs, which facilitate their spread through the surrounding neuronal cells. The involvement of EVs in the pathogenesis of ALS and other neurodegenerative diseases also arises through EV-carried miRNAs (including myomiR-218, -206, -133a, -133b, -1), regulating the establishment of the appropriate skeletal muscle–nerve interaction. An impaired expression of these EV-miRNAs has been observed during the onset of neurodegenerative diseases.

Altogether, these data suggest that presynaptic boutons are capable of releasing exosomes that are taken up by muscle cells, and that these exosomes have a physiological function in synaptic plasticity in the recipient muscle cell in vivo. In this anterograde pathway, exosome-carried Wg binds to postsynaptic Fz2, activating a signal cascade which leads to the differentiation of the postsynaptic membrane [54]. Wg also guides an autocrine response on the motoneuron; the binding of Wg with the heterodimer Fz2/Arrow activates the kinase Gsk-3β/Shaggy/Sgg, leading to cytoskeletal remodeling and bouton growth [55] (Figure 1A).

Altogether the reported evidence shows that Wnts are prominent factors in the regulation of synaptic development and plasticity; moreover, the EV-mediated release of Wnts seems to be a widespread mechanism. As a matter of fact, Wnts can be internalized as well as secreted via microglia, as documented by Kerr et al. [56], who showed that glia-derived Wg also regulates the localization of glutamate receptors at postsynaptic sites of the NMJ.

Continuous coordination of synaptic growth, in relation to muscle size, also requires the release of a retrograde signal of the BMP family that acts on BMP receptors in the presynaptic cell [57]. Currently, there is not direct evidence available on the role of EVs in the transport of BMPs in the NMJ. However, it is conceivable their involvement in the establishment and modulation of the NMJ because other experimental models showed that BMPs are carried by EVs [58]. Similarly, Clayton et al. [59] showed that TGF-β associated with EVs mediated anti-proliferative effects on blood lymphocytes.

Moreover, EVs seem to be involved in the transport of synaptotagmin 4 (Syt4, a membrane-trafficking protein), an essential retrograde signal from muscle to motoneuron. Since presynaptic boutons are capable of releasing exosomes containing Syt4 that are taken up by muscle, the Syt4 exosomal transport suggests the presynaptic control of retrograde signals [52].

## 4. Role of miRNAs in Motoneuron Function

A growing body of research has established that miRNAs play a major role in a wide range of developmental processes such as cell proliferation, apoptosis, developmental timing, neuronal cell fate [60,61,62,63,64], neuronal gene expression [65], brain morphogenesis [66], muscle differentiation [67] and stem cell division [68]. MicroRNAs have a key role in the fine-tuning of eukaryotic gene expression at the post-transcriptional level, inhibiting protein translation or promoting mRNA degradation, although in some cases they were found able to increase gene expression [69].

In the last decade, compelling evidence has also documented a crucial role for miRNAs in motoneuron development, function, survival and regeneration after injury [70]. In motoneuron progenitor cells, the DICER loss of function (an important component for miRNA biogenesis) causes aberrant development, as well as progressive motoneuron degeneration in adult mice [71]. For example, the dysregulation of miR-9 and miR-17-3p has been associated with negative consequences in the development and differentiation of chick embryo spinal motoneurons [72]. Although there is no knowledge of the complete miRNome of motoneurons at present, it is almost evident that there is a highly dynamic network of a subset of miRNAs, named MotomiRs, that is critical for the biology of motoneurons (development, viability and regeneration) [73]. Among the MotomiRs, only miR-218 has been described as a motoneuron-enriched miRNA; in more detail, miR-218 is crucial to establishing the right motoneuron identity regulating neuromuscular synapses, membrane excitability and motoneuron survival [74]. Indeed, miR-218 has been predicted to target and regulate 333 different motoneuron mRNAs, named TARGET^218^. Among these, the glutamate re-uptake receptor (SLC1A2) was found to be upregulated after miR-218 suppression [74], suggesting its essential role in preventing neuronal excitotoxicity, a fundamental event in motor neurodegeneration. MiRNAs are distributed in neuronal compartments where can regulate mRNA translation at axonal compartments. In particular, they seem to play a significant role at the NMJ. The main studies have been performed in *Drosophila* models, wherein it has been demonstrated that the lack of expression of miR-124, miR-125 and let-7 causes defects in NMJ function [75,76] and phenotype [77]. Other miRNAs, such as miR-8, miR-289 and miR-958, are involved in synaptic growth at the NMJ [78]. Moreover, the cluster miR-310/313, as well as miR-153, are involved in the regulation of synaptic homeostasis; in fact, they control the neurotransmitter release in motoneurons during the larval stage [79].

In light of accumulating evidence demonstrating that miRNAs are secreted enwrapped in EVs and that vesicles represent a novel form of information exchange within the nervous system [80], the above-reported data open a new exciting scenario for the involvement of extracellular miRNAs in NMJ establishment and function.

## 5. Impairment of Motoneuron–Muscle Communication Mediated by EV-miRNAs

Motoneuron degeneration is the leading candidate as the cause of neurodegenerative disorders [81], in which structural, physiological and metabolic changes of different cell types (such as motoneuron, muscle and Schwann cells) seem to contribute mutually and synergistically to the onset and severity of the disease. Many factors seem to lead to motoneuron death; for example, oxidative stress, excitotoxicity, deficits in axonal transport, mitochondrial dysfunction and miRNA dysregulation have been considered possible contributors. Among the cell types involved in this scenario, skeletal muscle is now considered an important tissue in the pathogenesis of motoneuron disorder activating a retrograde signaling cascade that contributes to motor neuron degradation [82]. The findings that muscle cells release in vitro [83] and in vivo [84] exosomes containing miRNAs [84,85] are in agreement with the idea that EV-miRNAs can function as signaling molecules at the NMJ.

Consequently, in pathological conditions miRNA cargo can change, thus modifying autocrine and paracrine signaling pathways [86]. Interestingly, there is a large body of evidence [87] supporting the role of miRNA dysregulation in degenerative disorders, such as amyotrophic lateral sclerosis (ALS) and spinal muscular atrophy (SMA) type 1.

Recently, in ALS model rats, miR-218 was found to be released extracellularly from motoneurons and taken up by astrocytes, downregulating the excitatory glutamate transporter 2 (EAAT2) [88]. Moreover, Rizzuti et al. [89] identified 15 downregulated miRNAs in motoneuron progenitors derived from human ALS-induced pluripotent stem cells and found that the predicted target genes of the differentially expressed miRNAs are involved in neurodegeneration-related pathways. The involvement of muscle in the pathogenesis of ALS is suggested by the dysregulation of muscle-specific miRNAs (myomiR) found in G93A-SOD1 transgenic mice, a murine model of ALS, and in the skeletal muscle of ALS patients. MyomiRs play an important role in the regulation of muscle homeostasis (proliferation, differentiation and regeneration). However, in addition to their canonical role, they are now considered important players in ALS pathogenesis due to their involvement in NMJ maintenance and repair. Among the canonical MyomiRs (miR-1, -133a/b and -206), miR-206 was found to be upregulated in the synaptic region of G93A-SOD1 mice and in the skeletal muscle of ALS patients, and seems to promote a partially successful compensatory response to skeletal muscle denervation, a common event during disease progression.

Moreover, G93A-SOD1 mice are characterized by strong oxidative stress; this evidence is consistent with the finding that H_2_O_2_-stressed myotubes release increased levels of exosomes carrying DNA which can induce inflammation [90]. In response to denervation, MyoD and myogenin promote the transcriptional activation of miR-206, which in turn targets HDAC4 (histone deacetylase 4) and FGF (fibroblast growth factor) signaling pathways in muscle. HDAC4 is a regulator of neuromuscular-related gene expression and muscle remodeling, influencing the formation of appropriate nerve types which connect to the muscle [91]. In fact, its inhibition induces the expression of FGFB1, a promoter of re-innervation and regeneration within NMJ. MiR-206 also regulates satellite cell markers, probably in an attempt to promote satellite cell activation, proliferation and differentiation required for myogenesis, as well as to assist with NMJ protection.

Following denervation, MyoD also promotes the expression of miR-133b, which is located in a bicistronic transcript together with miR-206 [92]. Interestingly, miR-133b directly stimulates neurite outgrowth following nerve damage in rat brain, suggesting its involvement in nerve regeneration; furthermore, miR-133b may suffice to replace absent functions in miR-206 null mice. These various observations imply the likelihood that both miR-206 and miR-133b have functions in the recovery and maintenance of nerve–muscle signaling. This is consistent with the finding that EV-MyomiRs are upregulated during muscle cell differentiation [85] and in response to muscle contraction [78].

On the contrary, miR-1 and miR-133a are downregulated following nerve injury; studies performed on *Drosophila* and *C. elegans* showed that miR-1 regulates both pre- and postsynaptic NMJ behavior, modulating the expression of nicotinic acetylcholine receptor (nAChR) subunits and thereby altering muscle sensitivity to ACh (Figure 1B).

It is noteworthy that other potential targets of miR-206 are BDNF, NGF and IGF-1, suggesting that mir-206 could also be involved in the regulation of muscle mass and synapse formation during re-innervation.

On balance, muscle EV-miRNAs may contribute to regulate developmental gene expression in growing muscle and peripheral nerve and aid in coordinating nerve and muscle gene expression programs in order to establish the appropriate skeletal muscle–nerve interaction and maintain a correct neuromuscular junction association. This idea is supported by recent findings in which a large increase in miR-206 was observed in exosomes produced by denervated myofiber, suggesting that its biological action could be not limited to local muscle repair but also extended to non-muscle neighboring cells, such as motor nerve terminals at the motor end plate [86].

Altogether, the above-reported data suggest that miRNAs encapsulated in EVs could participate and interfere with the balance between denervation/re-innervation and muscle regeneration/atrophy processes and can be regarded as important modulators of the course of neurodegenerative diseases.

## 6. Involvement of EVs in the Prion-Like Mechanism of Motoneuron Diseases

Recent data demonstrate the involvement of EVs in the transmission of misfolded proteins to surrounding cells, promoting the aggregation of other proteins in a prion-like mechanism [93,94,95] (Figure 1B). A growing body of evidence shows that amyotrophic lateral sclerosis is characterized by abnormal protein aggregation in motoneurons and neural accessory cells [95,96]. This pathology is a fatal neuromuscular condition characterized by the degeneration of upper and lower motoneurons, which causes progressive muscle paralysis and spasticity, affecting mobility, speech, swallowing and respiration [97]. ALS cases can be classified into two categories: sporadic ALS (SALS) and familial ALS (FALS).

SALS represents the 90–95% of all cases and is caused by some apparently facilitating gene mutations [98] such as repeated expansions of the gene that encodes ataxin-2 [99]. FALS occurs in mendelian-inherited mutations of the following genes: Cu/Zn superoxide dismutase (SOD1), TAR-DNA–binding protein 43 (TDP-43), fused in sarcoma/translocated in liposarcoma (FUS/TLS) and the chromosome 9 open reading frame 72 gene (*C9orf72*) [93,98].

These mutations induce protein misfolding, leading to the aggregation and formation of inclusion bodies. It is known that these aggregates induce cellular stress [100], giving rise to axonal retraction and ultimately cell death [98].

For example in the mutant SOD1 model of ALS, the intracellular aggregates of misfolded SOD1 escape proteasomal and autophagy degradation, accumulating intracellularly and leading to pathogenesis [98,100,101]. Moreover, it has been reported that mutant SOD1 can spread to the surrounding neural cells through macropinocytosis; once reaching the host cell, misfolded SOD1 rapidly exits the macropinocytic compartment and then acts as a seed inducing the aggregation of other SOD1 [102]. Recent evidence suggests that EVs are involved in this mechanism; indeed independent studies show that mutant SOD1 is secreted by neural cells packaged into EVs and, in this way, it can enter neighboring neural cells much more efficiently [93,95,103].

The reported prion-like mechanism appears not to be limited to SOD1, as suggested by TDP-43, FUS/TLS and C9orf72 examples.

TDP-43 is an RNA-binding protein containing a prion-like domain, which commonly shuttles between the nucleus and cytoplasm, where it lies in granules with mRNAs which are transcriptionally silenced [98]. In response to stressors, TDP-43 is detectable mainly in the cytoplasm, where it is incorporated into stress granules [98,104,105]. EVs have been shown to mediate the transfer of TDP-3 granules from cell to cell. Indeed, it has been observed that TDP-43 is secreted packaged in microvesicles [104] and exosomes [94]. Furthermore, in ALS patients, mutant TDP-43 packaged in exosomes seems to induce an increased activation of peripheral monocytes and an impairment in their cytokine secretion [106].

In a subset of FALS and SALS cases, pathogenesis causes were found in autosomal dominant mutations of the gene encoding for fused in sarcoma/translocated in liposarcoma (FUS/TLS) protein [107,108,109,110]. FUS is involved in DNA repair, splicing, transcription, dendritic RNA transport and miRNA biogenesis and function [107]. Similar to TDP-43, FUS is an RNA-binding protein that moves between the nucleus and the cytoplasm, participating in nucleo-cytoplasmic RNA shuttling [111]. Mutations in FUS cause an imbalance in the nucleo-cytoplasmic shuttling process, leading to its accumulation in the cytoplasm and the formation of aggregates consisting of FUS ribonucleoprotein complexes [112,113,114]. In this way, FUS gains toxicity and induces severe defects in synaptic transmission at the NMJ [115,116]. In ALS patients, like for SOD1 and TDP-43, EVs can carry mutant FUS promoting the diffusion of the FUS-mediated toxicity by a prion-like mechanism [95,107] (Figure 1B).

Finally, *C9orf72* contains a hexanucleotide repeat expansion (HRE) known to be the commonest single genetic cause of ALS [117]. The predicted protein encoded by the *C9orf72* gene belongs to the highly conserved GDP–GTP exchange factors for RAB GTPases [98,117,118] which are involved in the formation of multivesicular bodies and fusion events. Increasing evidence suggests that mutations in *C9orf72* may be involved in ALS pathogenesis, inducing impairment in the vesicular trafficking and extracellular vesicle secretion [117].

## 7. Conclusions and Perspectives

Pioneering studies on NMJ establishment in *Drosophila* have paved the way for our understanding of the contribution of EVs to the delivery of Wnts from neural presynaptic to postsynaptic muscle membrane. This first evidence has been corroborated by independent experiments in vertebrates, confirming the ability of EVs to carry morphogens such as Wnts and BMPs. Moreover, recent data provide important clues about the role of EVs in spreading proteins containing prion-like domains and deregulated miRNAs, thus contributing to neurodegenerative diseases. Of note, the involvement of EVs in the transport of mutated proteins during disease suggests a possible use of vesicles as early and easily accessible biomarkers since they can be detected from plasma.

However, many outstanding questions relating to EV biology remain. For example, it is unclear whether morphogens and miRNAs are differentially loaded in specific EV subpopulations and whether there are molecular signatures that target EVs to specific recipient cells either in physiological or pathological conditions.

Answering the above-reported questions will provide a deeper understanding of the precise role of EVs in muscle–motoneuron communication and will allow the elucidation of the activation pattern of specific signaling pathways induced by EV cargo in a time- and concentration-dependent manner.
